# SYT12 is a novel oncogene that promotes thyroid carcinoma progression and metastasis

**DOI:** 10.7150/jca.62555

**Published:** 2021-09-27

**Authors:** Lingli Jin, Danni Zheng, Danxiang Chen, Erjie Xia, Yaoyao Guan, Jialiang Wen, Adheesh Bhandari, Ouchen Wang

**Affiliations:** Department of Breast Surgery, The First Affiliated Hospital of Wenzhou Medical University, Wenzhou, Zhejiang, PR China.

**Keywords:** PTC, SYT12, biomarker, LNM, primary neoplasm focus type, histological type

## Abstract

**Background:** Thyroid malignancy is the most frequent endocrine malignant tumor whose incidence is still increasing. Mechanisms genomic variations play a major part in the pathogenesis of many types of malignancy. Synaptotagmin 12 (SYT12) is a member gene of the synaptotagmins family and SYT12's variants were shown to be associated with some malignancies. Nevertheless, SYT12's specific function and probable clinical value in papillary cancer were still unknown.

**Methods:** We conducted complete genome sequence of 39 pairs PTC malignant neoplasm and matched non-neoplastic tissues. We found that SYT12 was significantly overexpressed in thyroid malignancy. Next, we investigated the expression level of SYT12 and the relation between clinical information and SYT12 expression in thyroid cancer in the Cancer Genome Atlas (TCGA). QRt-PCR of else 40 pairs local verified cohort was performed to confirm the sequencing data and TCGA cohort. Then, we used small interfering RNA (si-RNA) to knock down the expression of SYT12 in PTC cells. Finally, proliferation, cell colony formation, migration, invasion, and apoptosis assays were done to demonstrate the function of SYT12.

**Results:** SYT12 is significantly overexpressed and higher expression of SYT12 upsurges the risk of lymph node metastatic and incidence rate of primary neoplasm multivariate focus type and classical histological type for PTC patients in TCGA cohort. *In vitro* experiments, the results of functional assays presented that knock-down of SYT12 inhibited the cell proliferation, cell colony formation, trans-well migration, and trans-well invasion and promoted cell apoptotic in PTC cell lines.

**Conclusion:** SYT12 was a novel oncogene that promotes thyroid carcinoma progression and metastasis potential and a potential biomarker for diagnosis and treatment in PTC.

## Introduction

Thyroid carcinoma is the most frequent endocrine malignant tumor worldwide. According to the United States cancer statistics, 52890 lately assessed identified cases and 2180 expected deaths in 2020 1. Except for morbidity number of thyroid malignancies gradually increase, the average age of morbidity crowd also become younger. Thyroid cancer cases upsurge by about 3% yearly amongst those whose age were 20-39 and 4% amongst those whose age were 20-39 2. Thyroid cancer was divided into many types. And among those papillary thyroid cancer (PTC) is the most frequent generic pathological pattern of thyroid malignant tumor, making up almost 80% of thyroid cancers. In contrast to the rising occurrence of thyroid malignancy, the mortality rate of thyroid cancers has remained stable the same period despite 3-6. And the estimated overdiagnosis rates for thyroid cancers range from 50% to 90% of newly diagnosed cases in women, under the influence of different regions and health care environment 4. Hence, an appropriate and precise diagnosis is essential to reducing the overtreatment of nonfatal thyroid cancers.

With the increasing knowledge of the molecular, it was recognized that mechanisms genomic variations play a major role in the pathogenesis of many types of malignancies, involving point mutations in chromosomal rearrangements and proto-oncogenes. MAPK activation could accelerate PTC initiation and progression and the mutations of BRAF V600E could activate MAPK. The activation of PI3K/AKT passage is also deeded be significant for initiation of thyroid cancer. he genetic mutation of RAS, PI3CA, AKT and PTEN all could stimulate this pathway 7. Despite the significant progress made in genetic research, the mechanisms underlying PTC tumorigenesis remain unclear and further research needs to be done to discover a more precise forecast modal.

Synaptotagmin 12 (SYT12) is a member of the synaptotagmin (SYT) family. As we all know, the synaptotagmin family regulated the release of neurotransmitters according accommodating the fusion of calcium-dependent synaptic vesicle membrane proteins. There is an N-terminal transmembrane region and a tandem C-terminal C2 domains in some SYTs 8. C2 domains serve as a connector connecting Ca2+. SYT1-3, 5-7 and 9 own C2 domains while SYT4, 8, and 11-16 do not 9.

In recent years, variants of the human SYT12 were shown to be associated with some cancers 10, 11. Liu et al reported that SYT12 acted as a possible oncogene by activating the PI3K-AKT-mTOR signal channel in lung adenocarcinoma 11. Keitaro et al also found that SYT12 played a crucial part in oral carcinoma and might be an innovative therapeutic target 10. And Jonklaas et al. showed that SYT12 may contribute to predicting PTC results in a prospective cohort, but they did not verify the specific biological functions by biology empirical study 12. In brief, the function of the SYT12 gene play in the beginning and progression of thyroid malignancies is still keeping mysterious. Therefore, a further research of the biological purposes about the human being SYT12 is significant to comprehend PTC tumorigenesis and evolution. The present investigation expected to discover the relationship amongst the expression level of human SYT12 and clinical descriptions, and observe the SYT12's biological property by utilizing small interfering RNA (si-RNA) in PTC cells.

## Materials and Methods

### Patients and Thyroid Tissue samples collection

We acquired 79 (39 pairs PTC malignant neoplasm and matched non-neoplastic tissues genetic for genetic sequencing of incomplete research project; 40 pairs PTC malignant neoplasm and matched non-neoplastic tissues genetic for real-time reverse transcription‑quantitative polymerase chain reaction) pairs PTC tumor and nearby non-neoplastic thyroid tissues from 79 PTC patients who were diagnosed by two pathologists at least. The inclusion criteria of those 79 PTC patients were as described below: (1) Having surgery in Department of Thyroid and Breast Surgery, The First Affiliated Hospital of Wenzhou Medical University, Wenzhou, Zhejiang, People's Republic of China; (2) one lymph node was examined at least (3) having complete clinical baseline features; (4) no preoperative radiotherapy; (5) no another malignant tumor was diagnosed before; (6) site recode according to the Third Edition of International Classification of Disease for Oncology (ICD-O-3): C73.9 Thyroid gland; (7) morphological recode according to ICD-O-3:8340/3 Papillary carcinoma, follicular variant; 8341/3 Papillary microcarcinoma; 8342/3 Papillary carcinoma, oxyphilic cell; 8343/3 Papillary carcinoma, encapsulated; 8344/3 Papillary carcinoma, columnar cell, and; (8) the stage according to the 7^th^ edition AJCC staging system: I-IV.

After resection, samples were put in liquid nitrogen as quickly as possible. If the samples were not used for RNA extraction immediately, they would be stored in -80 °C refrigerator. There was another cohort in our study and it was gained from the Cancer Genome Atlas Database (TCGA) database. In complete, we elected 502 PTCs and 58 non-neoplastic thyroid tissues with whole sequence data and complete clinical features. Each procedure was recorded with the ethical standards approval of the Ethics Committee of the First Affiliated Hospital of Wenzhou Medical University.

### RNA extraction, reverse transcription and real-time reverse transcription-quantitative polymerase chain reaction (RT-qPCR)

The genetic code data of 39 paired PTC samples and matched adjacent non-neoplastic thyroid samples were originated from another incomplete research project. We isolated the RNA from fresh tissues and cells by TRIzol (Thermo Fisher Scientific, Waltham, MA, USA). We inspected the quantity and quality of isolated RNA by A260/A280 ratio and spectrophotometric value. It was deemed as most efficient that 1 µg RNA per 20 µl system reacting contained. And a SYBR Premix Ex Taq II kit (RR820A, TaKaRa, Dalian, China) was used for Real-time reverse transcription‑quantitative polymerase chain reaction (qRt-PCR) on an applied Biosystems 7500 Real-Time PCR System. The mRNA expression comparing with GAPDH expression was calculated by the 2-ΔΔCt equation. All procedures were performed by standard instructions. The sequences of SYT12 primer were bought from Sangon Biotech (Shanghai, China), as described below: SYT12,5′-GCAACACCTTTGGGCAGGAC-3′ (forward) and 5′-GTGTGG GAGGCAGTGTCGTA -3′ (reverse). Each assay was performed in triplicate and the steps were performed by standard description.

### Cell lines and cell culture

Professor Mingzhao Xing of Johns Hopkins University School of Medicine (Baltimore, MD, USA) provided human PTC cell lines (TPC, KTC and BCPAP) which we needed. The Cell Bank of the Shanghai Chinese Academy of Sciences (Shanghai, China) offered the normal thyroid cell line (HTORI3) of human. All cells were cultivated in RPMI 1640 (Invitrogen; Thermo Fisher Scientific, Inc., Waltham, MA, USA) supplemented with 1X DMEM nonessential amino acids, 10% fetal bovine serum (FBS; Invitrogen; Thermo Fisher Scientific, Inc.) and 1X sodium pyruvate. And the nutrient solution containing cells was kept in incubator with CO2 concentration in 5% at 37 °C.

### siRNA Transfection

We knock-down the gene of SYT12 by small interfering RNA (siRNA) which was purchased by Gene Pharma (Shanghai, China). The siRNA sequences of SYT12 used in the present study were as follows:

siRNA1, forward 5'-GCAGAAUACCAUCUGAGCGTT-3' and reverse 5'-CGCUCAGAUGGUAUUCUGCTT-3'; siRNA2, forward 5'-UCAUCUGGACCAACGACAATT-3' and reverse 5'-UUGUCGUUGGUCCAGAUGATT-3'; siRNA3, forward 5'-CCAUCUUCUUUGAUGAGAATT-3' and reverse 5'-UUCUCAUCAAAGAAGAUGGTT-3'.

Those three siRNAs were all targeting to knock down the expression of SYT12, and different siRNA sequences had different knockout efficiency. Si-NC was offered by Gene Pharma as blank control when we bought the siRNAs. PTC cells were seeded into six-well plates (TPC 7×10^4^ cells/well; KTC 8×10^4^ cells/well) and cultivated for 24 hours before transfection. The expression of SYT12 was reduced by small interfering RNA (si-RNA) which was delivered to cells by a membrane destructive agent named RNAiMAX (Invitrogen, Grand Island, NY, USA) (si-RNA: RNAiMAX = 7.5 µl: 3 µl) according to the standard description. After 48 hours' of cultivating, cells were collected for following tests.

### Cell proliferation assay

Cell-counting kit 8 reagent (CCK-8, Beyotime, Biotechnology, Shanghai, China) was used to observe the proliferation ability of PTC cells. TPC and KTC cells which had been exposed in SYT12-siRNAs or NC-siRNA for 48 hours were seeded onto 96-well plates (1000 cells/plate). Then, the CCK-8 (10 µl/well) (Beyotime Biotechnology, Shanghai, China) was added to wells, and then the cells were incubated with at 37 °C for 3 hours. In the next four days, 450 nm absorbance was measured to drawn proliferation curves by spectrophotometer (DS-11 FX; DeNovix, Wilmington, USA). The experiment was repeated three times.

### Colony formation assay

After 48 hours transfection, the thyroid cancer TPC (1×10^3^ cells) and KTC cells (2×10^3^ cells) were plated into a 6-well plate. There were more than 50 cells in a colony formation, 8 days later. We fixed the cells by 4% paraformaldehyde for 30 minutes. Then, 0.1% crystal violet solution was used to stain the cells for 30 minutes. Those colonies which were more than 50 cells in a colony formation were counted and images were captured by digital camera. This was repeated three times. Altogether, experiments were achieved in triplicate.

### Migration and invasion assays

Transwell chambers (#3422, Corning, NY, USA) were used to measure the migration ability of PTC cells. TPC and KTC cells (3 × 10^4^ cells/well) which had been transfected for 48 hours were seeded into the upper chamber in 300 µl serum-free nutrient solution while the lower chamber contained 600 µl medium supplemented with 10% FBS. 22 hours later, the cells were fixed by 4% paraformaldehyde for 30 minutes. Then, 0.1% crystal violet solution was used to stain the cells for 30 minutes. We captured the pictures by microscope under the magnifying power of 10×40 for further analysis. BioCoat Matrigel Invasion Chambers (#354480, Corning Biocoat, USA) were used to exam the invasion ability and the procedures were similar to migration assay. The experiment was repeated three times.

### Apoptosis assays

Cell apoptosis ability was detected by Annexin V-fluorescein isothiocyanate (FITC) apoptosis kit (#556547; Becton, Dickinson and Company, Franklin Lakes, NJ, USA) with specification of manufacturer. After 48 hours transfection, cells were gathered and washed 3 times by phosphate-buffered saline (PBS). Then, we resuspended the cells into 1× binding buffer (1×10^6^ cells/ml). Next, cells were stained with Annexin V-FITC for 15 minutes and propidium iodide (PI) for 5 minutes in the dark before examined by flow cytometry (BD Biosciences Accuri C6; Becton, Dickinson and Company). And the results were analyzed by Flowjo software (Flowjo, Ashland, OR, USA). The experiment was repeated three times.

### Statistical analysis

All assays were repeated three times and data was shown as the mean ± SD. SPSS 25.0 software and GraphPad Prism 7.0 were the analytical software used to analyze the data. Student's t-test was used to evaluate two group comparisons and one‑way ANOVA was used to analyze multiple group comparisons. P < 0.05 was considered to be significant statistical difference.

## Results

### SYT12 is obviously overexpressed in PTC

**Figure 1:**
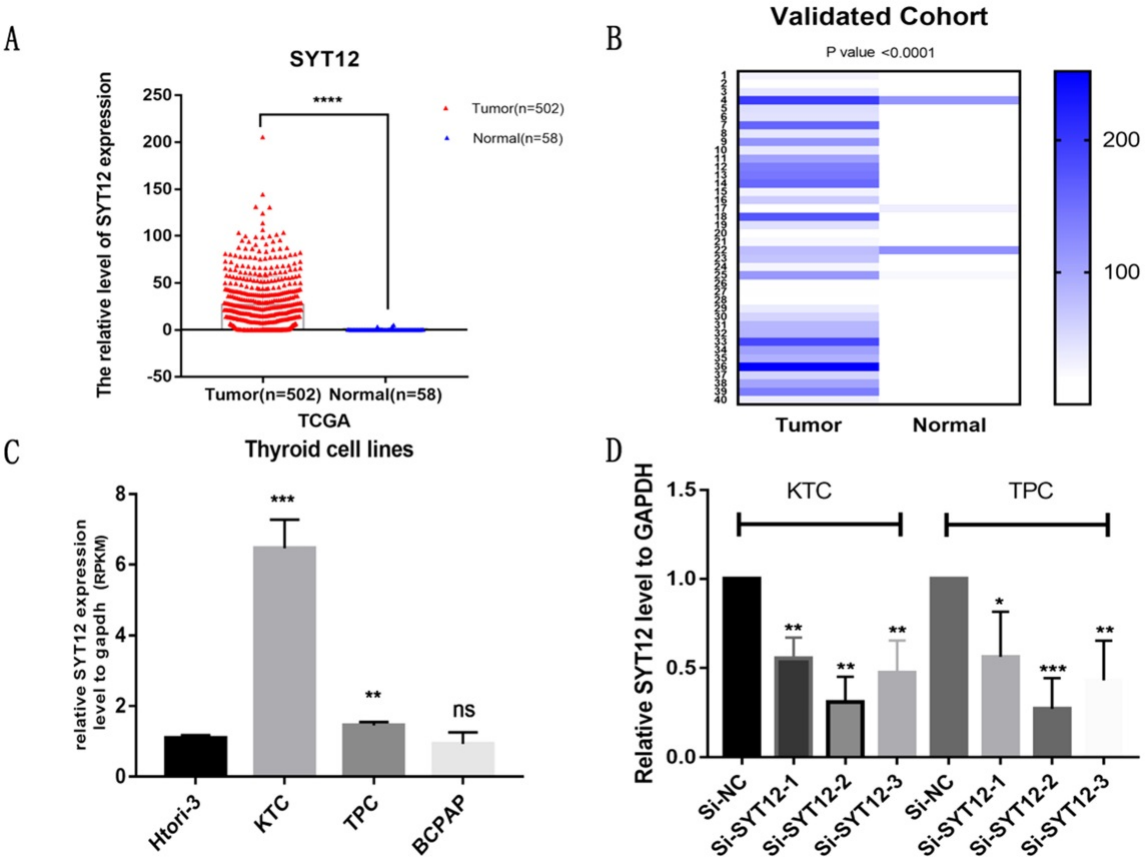
** SYT12 expression in thyroid cancer in the TCGA cohort. (A)** The mRNA expression level of SYT12 in PTC in TCGA cohort. **(B)** A hot map that describes the SYT12 expression examined by RT-qPCR in 40 paired thyroid cancer tissues and adjacent noncancerous tissues. **(C)** The relative expression of SYT12 in PTC cell lines. SYT12 was upregulated in two PTC cell lines (TPC and KTC) compared to normal thyroid cell line HTORI-3. **(D)** SYT12 expression levels of si-SYT12 groups and si-NC in the two PTC cell lines. * P<0.05, **p<0.01, ***p<0.001, ****p<0.0001.

To make an investigation of the function of SYT12 in PTC, we initially detected the expression level of SYT12 in 40 pairs tumor samples and nearby normal thyroid tissue via RT-qPCR. After analyzing this data, we identified that SYT12 expression in tumors was significantly upregulated. To describe the difference in our verified cohort, a heat map was made (40 pairs tumor tissues and matched non-cancerous tissues, ∆d = -66.36 ± 58.66, paired t-test, P < 0.0001) (Figure 1B). This result is consistent with the TCGA data (Tumor tissue 26.99 ± 1.321, n=502; Normal tissue 0.3156 ± 0.1181, n=58; unpaired t-test P <0.0001) (Figure 1A). Next, we test the mRNA expression of SYT12 in different thyroid cancer cell lines, the result is also the same as collected surgical tissue samples and TCGA cohort. The mRNA expression level of SYT12 in different PTC cell lines were obviously higher than non-neoplastic thyroid cell line, just like the trend of tissues (compared with HTORI‐3, KTC, P = 0.0003; TPC, P = 0.0082; BCPAP, P= 0.4684) (Figure 1C).

### Connection between the expression of SYT12 and clinical factors

To examine the part of SYT12 taking in the occurrence and development of PTC, we analyze the association between the expression level of SYT12 and clinical factors. And the whole patients were segmented into 2 sets (high expression group and low expression group), according to median value. We found that the histological type (P<0.001), primary neoplasm focus type (P=0.044), tumor stage (P=0.001), lymph node metastasis (P<0.001) and the disease stage (P<0.001) were obviously different in the 2 sets (Table 1) in TCGA cohort. But in the local cohort, we did not find the similar consequence as demonstrated in the TCGA cohort. 40 thyroid cancer tissues were analyzed in the local cohort (Table 2), there is no significant difference about primary neoplasm focus type (P=0.191), tumor stage (P=0.516), lymph node metastasis (P=0.324) and the disease stage (P=0.101). Probably because the volumes of local validated cohort were too small to draw a credible result.

### Higher expression of SYT12 increases the risk of the incidence rate of primary neoplasm multivariate focus type in patients with PTC

So as to examine the role of SYT12 expression take part in primary neoplasm multivariate focus type, we performed logistic regression. Univariate logistic regression analysis in TCGA cohort shown that the meaningful variables for primary neoplasm multivariate focus type were expression level of SYT12 (P=0.047), gender (P=0.002), histological type (P=0.002) and status (P=0.023) (Table 3). Multivariate logistic analysis presented that the expression level of SYT12 (P=0.008), gender (P=0.001), histological type (P=0.005) and status (P =0.021) were the significant variables for primary neoplasm multivariate focus type (Table 3). In short, upregulated expression of SYT12 serves as a risk of primary neoplasm multivariate focus type.

### Higher expression of SYT12 increases the risk of lymph node metastatic in patients with PTC

To further examine whether the level of SYT12 expression was a major influencing factor of lymph node metastatic, univariate and multivariate logistic regression analysis were done in TCGA cohort. Univariate logistic regression analysis in TCGA cohort confirmed that the crucial variables for the risk of lymph node metastatic were the expression level of SYT12 (P<0.001), age (P<0.001), histological type (P<0.001) and disease stage (AJCC7) (P<0.001) (Table 4). Multivariate logistic analysis displayed that the expression of SYT12 (P<0.001), age (P<0.001), histological type (P<0.001) and disease stage (AJCC7) (P<0.001) impacted the risk of lymph node metastatic (Table 4). That is, the upregulation of SYT12 aggrandized the risk of LNM.

### Higher expression of SYT12 increases the risk of the incidence rate of classical histological type in patients with PTC

We found the expression level of SYT12 is a significant risk factor for the risk of incidence rate of classical histological type by logistic regression. Univariate logistic regression analysis in TCGA cohort confirmed that the crucial variables for the risk of incidence rate of classical histological type were the expression of SYT12 (P<0.001), lymph node metastasis (P<0.001), primary neoplasm focus type (P=0.002), tumor stage (P=0.032), distant metastasis (P=0.033) and status (P=0.026) (Table 5). Multivariate logistic analysis showed the same result that the expression of SYT12 (P<0.001), lymph node metastasis (P<0.001), primary neoplasm focus type (P=0.002), tumor stage (P=0.003), distant metastasis (P=0.031) were connected with the risk of the risk of incidence rate of classical histological type. All the above suggested that overexpression of SYT12 increased the risk of incidence rate of classical histological type in PTC.

### Knock-down of SYT12 inhibited the proliferation of PTC cells

**Figure 2:**
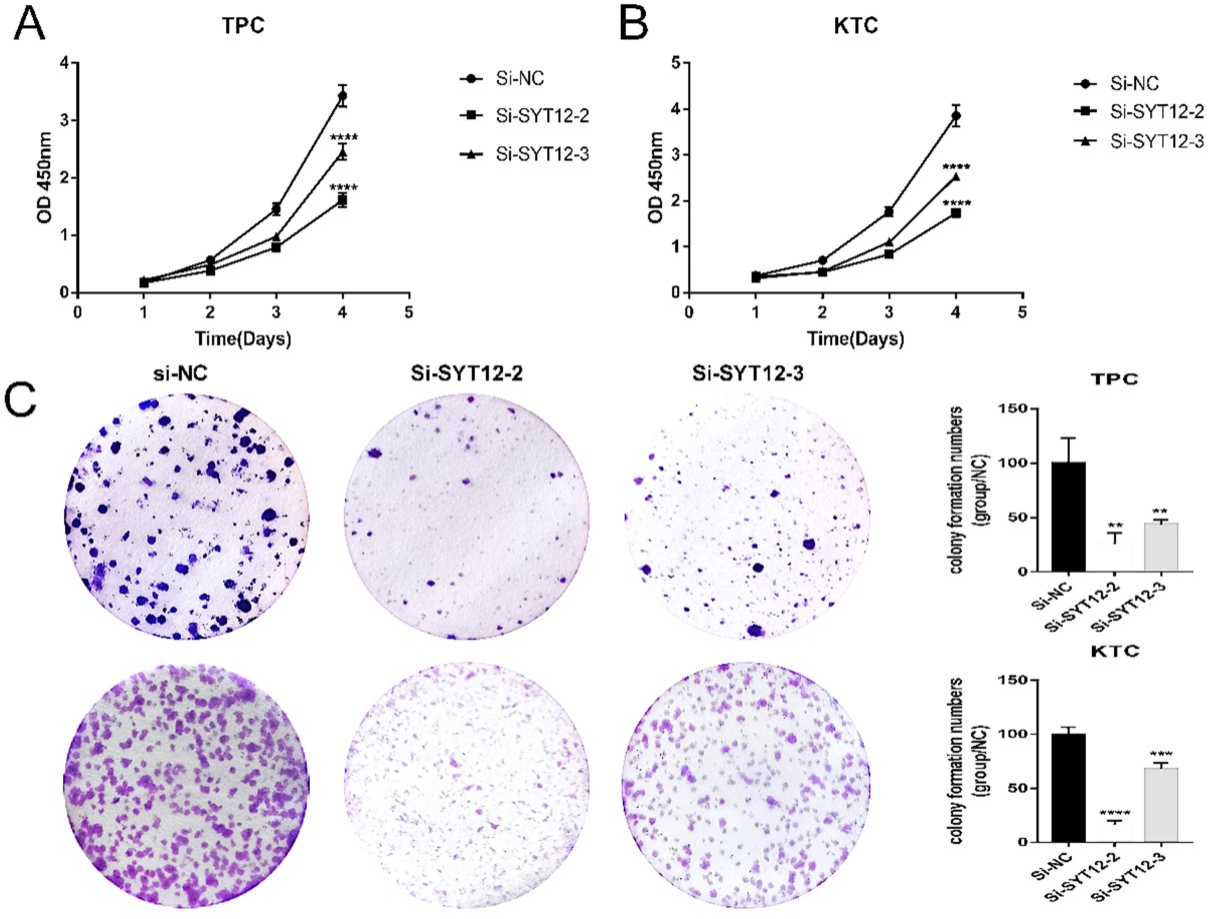
** Down-regulated SYT12 expression inhibited TPC and KTC cell proliferation abilities. (A)** CCK-8 assays performed in TPC cell lines. **(B)** CCK-8 assays were performed in KTC cell lines. **(C)** Colony formation assay in TPC and KTC cells and a corresponding number of colonies. * P<0.05, **p<0.01, ***p<0.001, ****p<0.0001.

The SYT12 gene is usually over-expression in PTC, it was assumed that SYT12 plays a significant role in tumorigenesis and progression in thyroid cancer. First, we test the expression of SYT12 in different PTC cell lines by RT-qPCR analysis. We found that this gene expression higher in KTC and TPC (Figure 1C). Therefore, we selected KTC and TPC as experimental cells. Similarly, effective siRNA2 and siRNA3 which considerably reduced the expression of SYT12 in the TPC and KTC cell lines (Figure 1D) for the following experiments. Whereafter, CCK-8 assays and cell colony formation assays were achieved. We discovered that knocked down the expression of SYT12 observably suppressed proliferation ability of PTC cell lines (KTC and TPC) (Figure 2).

### Knock-down of SYT12 inhibited the trans-well migration and invasion of PTC cells

**Figure 3:**
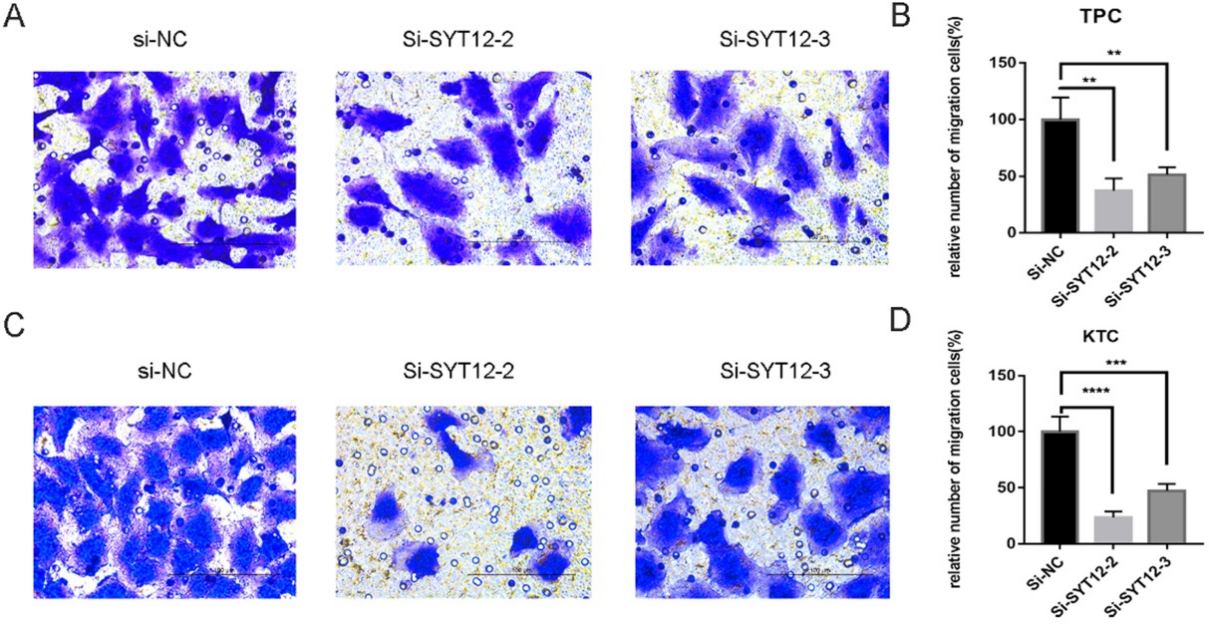
Down-regulation SYT12 gene expression in TPC and KTC cells inhibits migration. **(A, B)** Transwell migration assays in downregulation SYT12 cells and their corresponding control cells in TPC.** (C, D)** Transwell migration assays in downregulation SYT12 cells and their corresponding control cells in KTC. * P<0.05, **P<0.01, *** P<0.001, ****P<0.0001.

**Figure 4:**
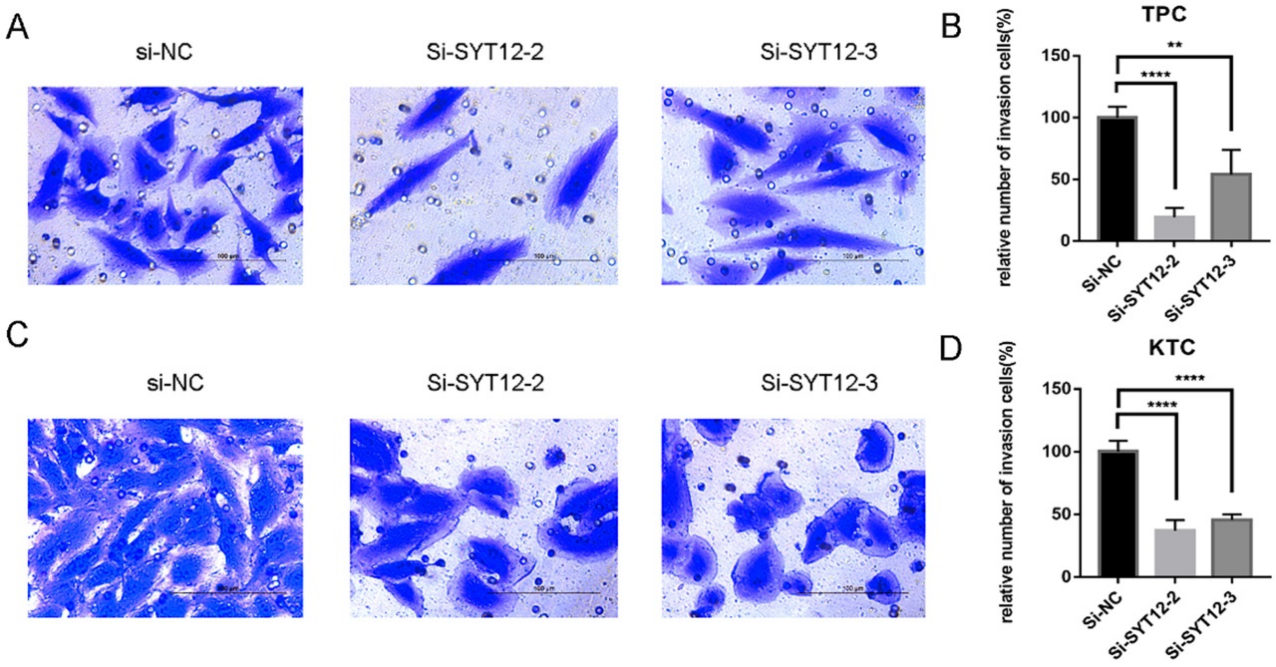
Down-regulation SYT12 gene expression in TPC and KTC cells inhibits invasion. Transwell invasion assays in downregulation SYT12 cells and their corresponding control cells in TPC. **(C, D)** Transwell invasion assays in downregulation SYT12 cells and their corresponding control cells in KTC. * P<0.05, **P<0.01, *** P<0.001, ****P<0.0001.

As is mentioned before the higher expression of SYT12 was correlated with LNM in the biological information analysis, thus we demonstrated the character of SYT12 in PTC by trans-well migration and trans-well invasion assays which often use to evaluate tumors metastasis capacity. The result of the cell migration assays indicated that number of wandering cells of TPC (Figure 3A & B) and KTC (Figure 3C & D) cells which shifted from upper chamber to the nether chamber exhibited a different result. The wandering cells which were transfected with si-SYT12s were significantly more than cells transfected with si-NC. The result of the trans-well invasion assays was the same as the migration assay (Figure 4). In a word, knock-down of SYT12 suppressed the TPC and KTC trans-well migration and trans-well invaded ability.

### Knock-down SYT12 promoted the cell apoptosis of PTC cells

**Figure 5:**
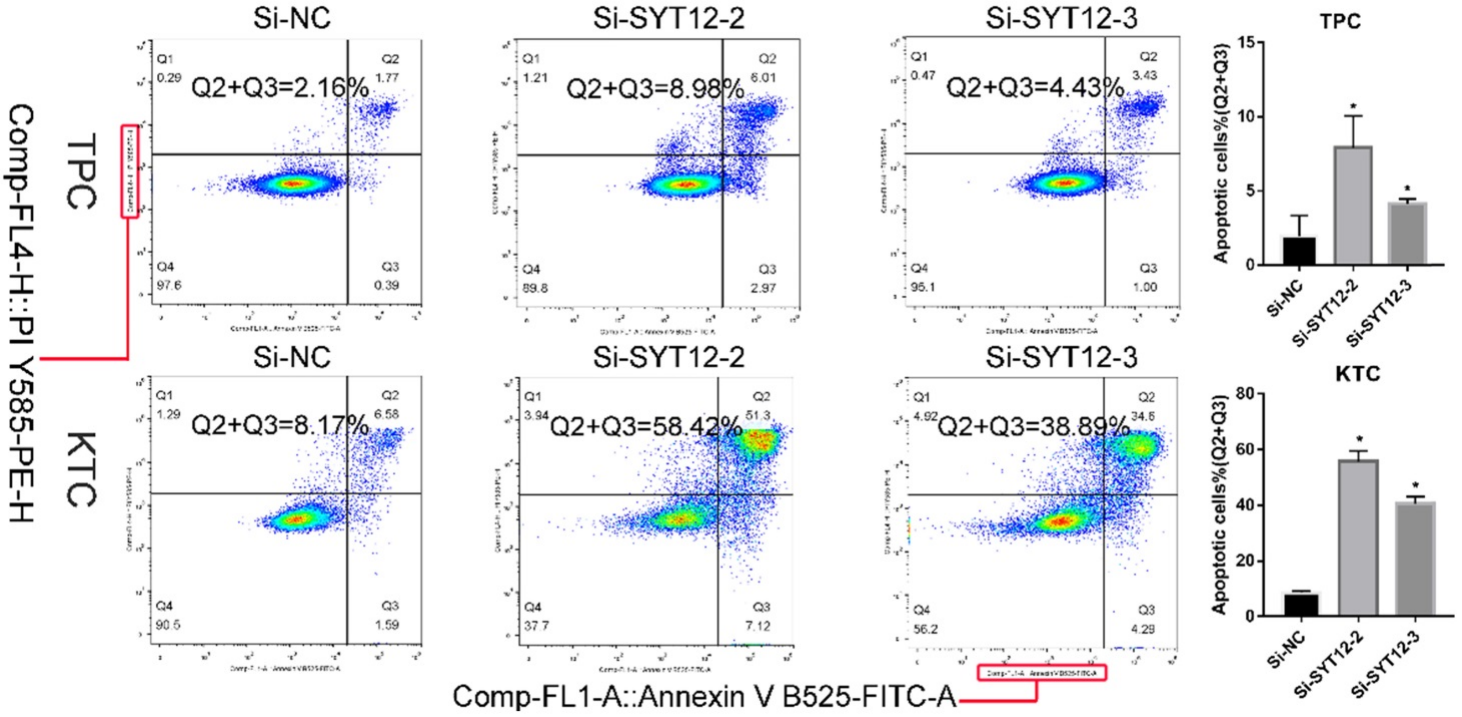
Down-regulation SYT12 gene promote apoptosis TPC and KTC cells. * P<0.05, **P<0.01, *** P<0.001, ****P<0.0001.

It is well-know that most tumor cells display a property, owing low apoptosis rate. To further probe the relationship between SYT12 expression and the tumorigenesis of PTC cells, apoptosis assays were done. We applied flow cytometry to measure cell apoptosis in TPC and KTC cell lines after transfection. Then, we quantified the results by the number of early apoptotic cells plus the late apoptotic cells. We discovered that knock-down SYT12 in KTC and TPC cell lines could promote cell apoptosis, especially in early apoptotic cells (Figure 5). In short, down-regulation SYT12 promoted the PTC cell apoptosis capacity.

## Discussion

Thyroid cancer has developed one of the most frequent malignant tumors in current years, and the morbidity number of thyroid malignancies also increased rapidly 13. At the same time, the patients who got thyroid malignancy were younger on average 2. Rahib et al. predicted that thyroid cancer would outstrip colorectal cancer by the year 2030 and develop the 4^th^ most frequently identified cancer 14. But the mortality rate of thyroid cancers has remained stable which is different from the increasing incidence of thyroid cancer in the same period 4-6. That is to say, the thyroid may be over-diagnosed at present. Furthermore, Bray et al. showed research that there was an estimated 50%-90% overdiagnosis rate for thyroid cancer with the influence of regions and health care environment 4. Although most patients with thyroid cancer had a good prognosis, there still were some thyroid cancer patients with local invasion or distant metastasis who more likely to appear recurrence or death 15. The overall survival rate of patients with partial recrudescence was about 70% to 85%, and the overall survival rate of patients with distant metastasis was 30% to 60% 16, 17. At present, surgeons make treatment decisions mainly relying upon the pathologic parameters and present clinical, which are inadequate to distinguish therapy and estimate the risk for different PTC patient 12. So, it is crucial to find a more effective way to differentiate low-risk thyroid malignant tumor patients and high-risk thyroid malignant tumor patients.

Thyroid malignancy is a greatly multifarious disease which can be divided into PTC, FTC, primary thyroid lymphoma, medullary thyroid cancer, anaplastic thyroid cancer and primary thyroid sarcoma 3. PTC is the most familiar kind of thyroid malignancy and its tumorigenesis is a very complex process 18. According to numerous previous studies, we realized that the tumorigenesis and progression of thyroid malignancy were mostly actuated by genomic mutation, comprising the silencing of contra-oncogenes and initiation of cancer-promoting genes 7, 19, 20. Between these genes, BRAF V600E was the very well-known 21, 22. However, the single BRAF V600E was not enough to predict PTC patients' prognosis 23, 24. These provided a new idea that if we could find novel molecular biomarkers combined with BRAF V600E, we might get a more efficient way to predict the risk of PTCs in clinical practice.

To discover a gene that associates with PTC that could differentiate low-risk and high-risk PTC patients, we send 39 paired PTC samples for RNA sequencing and discovered that SYT12 was overexpressed in PTC tissues associated with nearby non-neoplastic thyroid tissues. There are a few reports about SYT12, and it has been found that played a crucial role in some cancers such as oral cancer and lung adenocarcinoma 10, 11. Jonklaas et al. also showed that SYT12 could predict papillary thyroid cancer outcomes in a prospective cohort 12. But the part of the SYT12 gene taking in occurrence and development of thyroid tumor has yet remained unclear.

In the present analysis, we identified that SYT12 was obviously overexpressed in PTC tissues associated with normal tissues in the TCGA cohort according to the bioinformatics analysis. And it was consistent with the RT-qPCR analysis of collected 40 paired surgical tissue samples and thyroid cell lines. Also, it was shown that higher expression of SYT12 was a risk factor for LNM and increased the incidence rate of primary neoplasm multivariate focus type and classical histological type in patients with PTC by logistic regression in the TCGA cohort.

Loss-of-function assays presented that knocked down SYT12 could inhibit the proliferation, migration, colony formation, and invasion abilities of PTC cells *in vitro*. Furthermore, SYT12 knock-down could increase the apoptotic rate of PTC cells. These results were reliable with clinicopathological descriptions that SYT12 was related with LNM, primary neoplasm focus type, and histological type. In brief, SYT12 is an oncogene that promotes thyroid carcinoma progression and metastasis in PTC.

Nevertheless, our research has some limitations. Initially, the quantity of local samples is too small, and more cases are required to provide more strict results. Secondly, our assays were all *in vitro* and SYT12 function should be confirmed *in vivo* by additional animal model assays. Thirdly, the mechanism of SYT12 in PTC's occurrence and development yet remain unclear and compels additional study. Lastly, data of disease-free survival and overall survival lacked, thorough data collection is necessary for discussing the prognostic value of SYT12.

As a whole, our results indicate that SYT12 was up-regulation in primary PTC and its down-regulation could inhibit proliferation, migration, invasion, and facilitates cell apoptosis. This study showed that SYT12 was a probable biomarker for diagnosis and prognosis in PTC.

## Figures and Tables

**Table 1:** Association between the expression of SYT12 and clinicopathological factors in the TCGA cohort

Clinicopathologic factors	Patients	High expression (%)	Low expression (%)	χ^2^	p-value
**Gender**					
Female	128	64 (50)	64 (50)		
Male	360	182 (50.6)	178 (49.4)	0.012	0.914
**Age(years)**					
<45	229	111 (48.5)	118 (51.5)		
≥45	259	135 (52.1)	124 (47.9)	0.648	0.421
**Histological type**					
Classical	346	203 (58.7)	143 (41.3)		
Other types	142	43 (30.3)	99 (69.7)	32.459	<0.001***
**Primary neoplasm focus type**					
Unifocal	264	122 (46.2)	142 (53.8)		
Multifocal	224	124 (55.4)	100 (44.6)	4.054	0.044*
Tumor stage					
I+II	296	131 (44.3)	165 (55.7)		
III+IV	192	115 (59.9)	77 (40.1)	11.394	0.001**
**Lymph node metastasis**					
No	272	104 (38.2)	168 (61.8)		
Yes	216	142 (65.7)	74 (34.3)	36.436	<0.001***
**Disease stage (AJCC7)**					
I+II	323	143 (44.3)	180 (55.7)		
III+IV	165	103 (62.4)	62 (37.6)	14.394	<0.001***
**Distant metastasis**					
No	479	242 (50.5)	237 (49.5)		
Yes	9	4 (44.4)	5 (55.6)	0.131	0.718
**New event**					
No	444	226 (50.9)	218 (49.1)		
Yes	44	20 (45.5)	24 (54.5)	0.475	0.491
**Status**					
Alive	472	239 (50.6)	233 (49.4)		
Dead	16	7 (43.8)	9 (56.2)	0.294	0.588

Notes: *p-value< 0.05, **p-value<0.01, ***p-value<0.001; Abbreviations: SYT12, synaptotagmin 12; AJCC7, American Joint Committee on Cancer 7^th^ edition.

**Table 2:** Association between the expression of SYT12 and clinicopathological factors in the validated cohort

Clinicopathologic factors	Patients	High expression (%)	Low expression (%)	χ^2^	p-value
**Gender**					
Female	26	13 (50)	13 (50)		
Male	14	6 (42.9)	8 (57.1)	0.186	0.666
**Age (years)**					
<45	26	13 (50)	13 (50)		
≥45	14	6 (42.9)	8 (57.1)	0.186	0.666
**Primary neoplasm focus type**					
Unifocal	31	13 (41.9)	18 (58.1)		
Multifocal	9	6 (66.7)	3 (33.3)	1.711	0.191
**Tumor stage**					
I+II	19	8 (42.1)	11 (57.9)		
III+IV	21	11 (52.4)	10 (47.6)	0.422	0.516
**Lymph node metastasis**					
No	18	7 (38.9)	11 (61.1)		
Yes	22	12 (54.5)	10 (45.5)	0.973	0.324
**Disease stage (AJCC7)**					
I+II	34	18 (52.9)	16 (47.1)		
III+IV	6	1 (16.7)	5 (83.3)	2.691	0.101

Notes: *p-value< 0.05, **p-value<0.01, ***p-value<0.001. Abbreviations: SYT12, synaptotagmin 12; AJCC7, American Joint Committee on Cancer 7^th^ edition.

**Table 3:** Univariate and multivariate logistic regression analysis for the primary neoplasm focus type in the TCGA cohort

Clinicopathologic factors	Univariate analysis			Multivariate analysis		
	OR	95% CI	P-value	OR	95% CI	P-value
STYT12 Expression (high vs. low)	1.506	1.006-2.253	0.047*	1.681	1.145-2.470	0.008**
Gender (female vs. male)	0.499	0.324-0.769	0.002**	0.488	0.321-0.744	0.001**
Age (>45 vs. <45)	1.143	0.682-1.916	0.611		-	
Histological type (classical vs. others)	2.007	1.291-3.120	0.002**	0.55	0.361-0.838	0.005**
Lymph node metastasis (yes vs. no)	1.467	0.943-2.281	0.089		-	
Tumor stage (III, IV vs. I, II)	1.031	0.651-1.635	0.896		-	
Disease stage (AJCC7) (yes vs. no)	1.079	0.572-2.035	0.815		-	
Distant metastasis (yes vs. no)	0.121	0.014-1.041	0.054		-	
New event (yes vs. no)	0.793	0.400-1.571	0.506		-	
Status (dead vs. alive)	0.168	0.036-0.785	0.023*	0.169	0.037-0.762	0.021*

Notes: *p-value< 0.05, **p-value<0.01, ***p-value<0.001; Abbreviations: SYT12, synaptotagmin 12; AJCC7, American Joint Committee on Cancer 7^th^ edition.

**Table 4:** Univariate and multivariate logistic regression analysis for the lymph node metastatic risk in the TCGA cohort

Clinicopathologic factors	Univariate analysis			Multivariate analysis		
	OR	95% CI	P-value	OR	95% CI	P-value
STYT12 Expression (high vs. low)	2.334	1.500-3.631	<0.001***	2.328	1.514-3.579	<0.001***
Gender (female vs. male)	0.67	0.407-1.104	0.116		-	
Age (>45 vs. <45)	0.031	0.009-0.102	<0.001***	0.031	0.010-0.103	<0.001***
Histological type (classical vs. others)	2.896	1.721-4.874	<0.001***	2.645	1.615-4.331	<0.001***
Primary neoplasm focus type (Mul vs. Uni)	1.558	0.996-2.436	0.052		-	
Tumor stage (III,IV vs. I,II)	0.971	0.590-1.598	0.909		-	
Disease stage(AJCC7) (yes vs. no)	56.886	16.118-200.769	<0.001***	60.017	17.997-200.145	<0.001***
Distant metastasis (yes vs. no)	0.756	0.164-3.491	0.72		-	
New event (yes vs. no)	1.592	0.771-3.289	0.209		-	
Status (dead vs. alive)	0.978	0.269-3.563	0.974		-	

Notes: *p-value< 0.05, **p-value<0.01, ***p-value<0.001; Abbreviations: SYT12, synaptotagmin 12; AJCC7, American Joint Committee on Cancer 7^th^ edition.

**Table 5:** Univariate and multivariate logistic regression analysis for the histological type in the TCGA cohort

Clinicopathologic factors	Univariate analysis			Multivariate analysis		
	OR	95% CI	P-value	OR	95% CI	P-value
STYT12 Expression (high vs. low)	3.461	2.183-5.487	<0.001***	3.314	2.103-5.224	<0.001***
Gender (female vs. male)	0.834	0.503-1.383	0.483		-	
Age (>45 vs. <45)	0.806	0.461-1.411	0.451		-	
Lymph node metastasis (yes vs. no)	2.669	1.610-4.425	<0.001***	2.773	1.726-4.457	<0.001***
Primary neoplasm focus type (Mul vs. Uni)	0.494	0.317-0.769	0.002**	0.494	0.320-0.764	0.002**
Tumor stage (III, IV vs. I, II)	0.544	0.312-0.948	0.032*	0.501	0.316-0.794	0.003**
Disease stage (AJCC7) (yes vs. no)	0.846	0.312-0.948	0.652*		-	
Distant metastasis (yes vs. no)	0.163	0.031-0.861	0.033*	0.178	0.035-0.917	0.039*
New event (yes vs. no)	1.291	0.584-2.857	0.528		-	
Status (dead vs. alive)	11.792	1.350-103.025	0.026*	10.763	1.245-93.045	0.031*

Notes: *p-value< 0.05, **p-value<0.01, ***p-value<0.001l Abbreviations: SYT12, synaptotagmin 12; AJCC7, American Joint Committee on Cancer 7^th^ edition.
